# Supplementary material for: Confidence controls perceptual evidence accumulation
Source: Nat Commun. 2020 Apr 9;11:1753. doi: 10.1038/s41467-020-15561-w (PMC7145794; doi:10.1038/s41467-020-15561-w)
Supplement: Supplementary file 1 — Supplementary Information [file 41467_2020_15561_MOESM1_ESM.pdf]

Supplementary Information

Confidence controls perceptual evidence accumulation

Balsdon et al.

*correspondence: tarryn.balsdon@ens.fr

Supplementary Note 1

Our model assumed that, in the stopping task, observers entered their response when they thought they had accumulated enough evidence to meet the target performance on each trial, as they were instructed to do. We gave observers feedback on their average performance over sub-blocks of 20 trials. Observers could have tried to meet the target performance using a different strategy, for example, aiming for 100% accuracy on the first 14 trials, and then 0% accuracy on the next six trials (to achieve 70% correct), or aiming for 100% accuracy on the first 10 trials and then guessing (50% accuracy) on the next 10 (to achieve 75% correct). Or more simply, observers who felt as though they performed too well throughout the majority of the sub-block could start making errors, or responding after very few samples, in order to lower their accuracy. If this were the case, there would be a difference in accuracy across the block, and possibly a difference in the number of samples. There was no evidence for this, as shown in Supplementary Figure 1a. We instructed observers to respond when they thought they had a certain probability of a correct response on each trial, in other words, employ a flat bound on proportion correct. A flat bound on proportion correct would mean that the probability of a correct response should be independent of the number of samples: there should be no difference in the distribution of correct and incorrect responses over the number of samples. Supplementary Figure 1b (top) shows these distributions are indeed closely matched. Further, an observer who employs a non-stationary decision policy, where they purposefully make some guesses, would probably show fewer samples for incorrect responses on average (a guess can be made after a single sample, and is more likely to result in an incorrect response). No observer showed a substantial change in the number of stimuli for correct and error responses (Supplementary Figure 1b, bottom), and at the group level there was no significant difference in the number of samples for correct and incorrect responses, except in the 85% correct condition, where there was actually an increase in the average number of samples for incorrect responses (of approximately one sample on average p_{bonf*3} = 0.01).

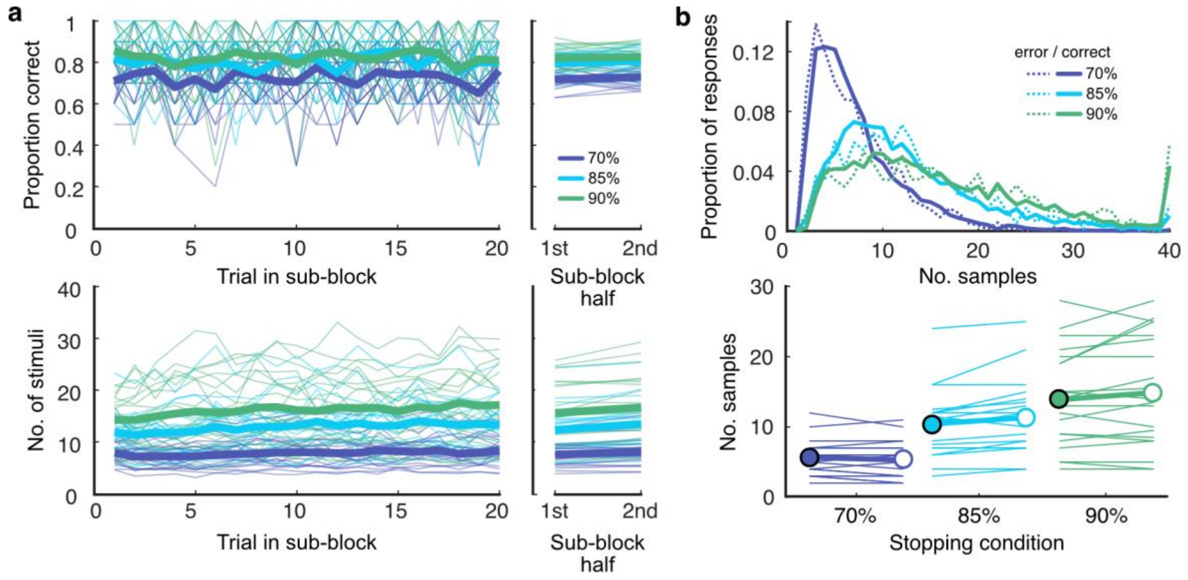

Supplementary Figure 1. Behaviour within sub-blocks. a) *Top left: proportion correct by trial number in sub-block. Fine lines show individual subjects (10 trials per data-point), thick lines show average across subjects. Top right: proportion correct in the 1st and 2nd half of the sub-blocks. At the extreme, to obtain 70% correct using a deviant strategy, an observer would need to be at 100% in the first half, and 50% in the second half. Bottom left: average number of samples by trial in sub-block. Bottom right: number of samples by 1st and 2nd half of the sub-blocks. At the extreme, to obtain 70% correct using a deviant strategy an observer would need to look at around 15 samples in the first half, and just one sample in the second half.* ***b)*** *Top: proportion of correct responses by number of samples (solid) and proportion of incorrect responses by number of samples (dashed), colours show the different conditions in the Stopping task. Bottom: median number of samples for correct and incorrect responses for each participant (thin lines) and averaged across participants (thick lines; correct responses shown with filled markers, incorrect with empty), for each condition in the Stopping task.*

Supplementary Note 2

In the Replay task, we asked observers to rate their confidence that they made a correct decision in order to measure their ability to estimate the validity of their own perceptual judgements (Type-II efficiency). The accuracy of these ratings is based both on the observer's insight into their Type-I accuracy, and how well they set criteria for making the confidence ratings. Whilst the former reflects Type-II sensitivity, the latter can be corrupted by bias – an observer may be more or less willing to give a high confidence rating. Therefore, care needs to be taken when estimating Type-II efficiency from confidence ratings. Type-II efficiency was calculated using the confidence ratings in the Replay task by calculating meta-d' as described in[1], and dividing by d'. We had originally planned to compare Type-II efficiencies between conditions. However, there were no significant between-condition differences (Kruskal-Wallis χ^2 = 1.83, p = 0.40), as shown in Supplementary Figure 2. This was likely due to the high uncertainty in the obtained estimates, as there were only 100 trials to calculate each observers' meta-d' in each condition. In subsequent analyses, we therefore used efficiency estimates computed using data pooled across all conditions. We could therefore only analyse between-subject differences, as opposed to within-subject comparisons between conditions.

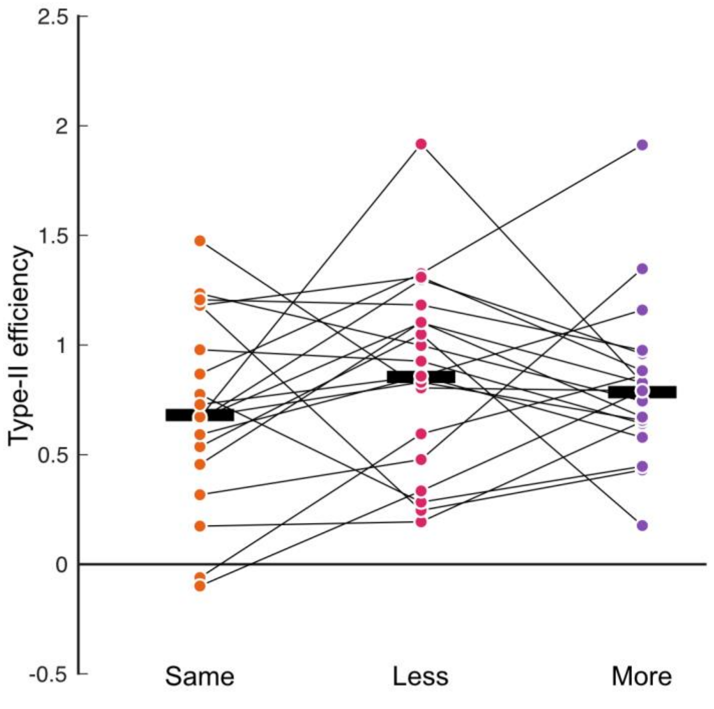

Supplementary Figure 2. Type-II efficiency for the Same (left), Less (middle) and More (right) conditions. *Thick horizontal bars show the mean, with each subject (n=20) marked by three connected dots across conditions. Type-II efficiency estimates showed no significant differences between conditions.*

Supplementary Note 3

A core aspect of making the Type-I decision is the setting and maintenance of the decision bound. Often it is sufficient to assume a flat bound over accumulation time, where the observer makes their decision after a certain quantity of evidence has accumulated, irrespective of the time it takes to accumulate this evidence. However, in our experiment we sought to understand how human observers were setting their bounds relative to the optimal observer, in order to measure their efficiency in setting and maintaining Type-I decision bounds. The optimal observer sets a flat bound on the likelihood of a correct response, which is not necessarily a flat bound on the total accumulated evidence. Thus, to understand how human observers should set their decision bounds to meet performance targets in the Stopping task, we performed simulations of the optimal observer. The optimal observer is an observer who experiences suboptimal evidence accumulation but who has sufficient insight into their suboptimalities to appropriately set their decision bound to meet the performance targets. Taking an example set of samples – corresponding to the materials used for one of the actual participants - we simulated suboptimal evidence accumulation by corrupting the ideal evidence provided by each sample with 10,000 samples of noise (drawn from zero-mean Gaussian with standard deviation σ) and performing leaky (controlled by parameter α) evidence accumulation across successive samples, resulting in 10,000 simulations for each of the 600 trials. For every trial, the optimal observer commits to a decision (stops accumulating evidence) when the proportion of simulations where the predicted response (corresponding to the sign of the accumulated log-odds evidence) is correct reaches or exceeds the performance target. In this way, the optimal observer is assumed to have access to the probability of making a correct response based on the accumulated evidence with each new sample (something human observers did not have direct access to). Plotting the average accumulated evidence at decision time against the number of samples the optimal observer chose to respond to on each trial revealed a non-monotonic function whose shape was not obvious to model (as shown in Figure 2a in main text). However, plotting the average accumulated evidence at decision time divided by the number of samples (proportional evidence) against the number of samples showed a monotonically decreasing function. This function was well approximated by an exponential function, as shown in Supplementary Figure 3a. For comparison, worse fits were obtained when approximated by a power function, as shown in Supplementary Figure 3b. This demonstrates that the optimal bound on Type-I evidence accumulation is well approximated by an exponentially decreasing function of the proportional evidence, allowing us to describe the bound by a function with just three parameters (λ, a and b; Equation 9 in Methods), rather than the height of the bound at each sample (up to 40 parameters). Our modelling approach consisted in comparing human observers' behaviour to this optimal behaviour, where again, describing the bound as an exponential function meant that differences from the optimal bound could be summarised by specific parameters of the function.

Regarding Type-II decisions, additional criteria were necessary to describe how observers rated their confidence. These criteria are usually modelled as additional bound-like divisions (confidence criteria) on the accumulated evidence. Before assuming that confidence criteria followed the same function as the exponentially decreasing bound on Type-I evidence, we took a normative approach to examine whether human observers' behaviour could reasonably be described in this way. Supplementary Figure 3c shows the average proportional evidence (its ideal estimate, neither disrupted by noise nor leak) as a function of the number of samples, binned by confidence rating for three example observers. Though there was large between-observer variability in the setting of confidence criteria, the functions appeared to monotonically decrease with increasing number of samples and could still be reasonably approximated by an exponential function.

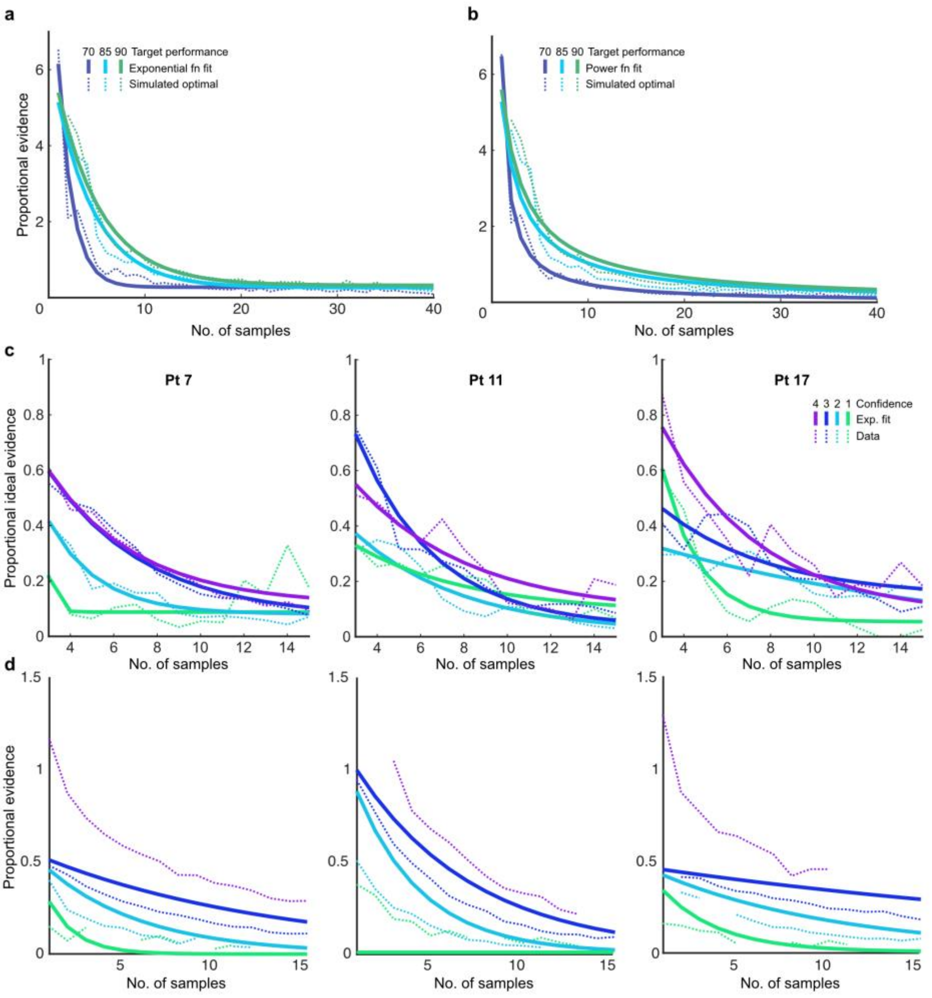

Supplementary Figure 3. Decision bounds and confidence criteria. a) *The y-axis shows the proportional evidence (disrupted by samples of noise and leak – in this case, $\sigma = 1.5$, $\alpha = 0.9$) which is the accumulated evidence divided by the number of samples the evidence was accumulated over. The dashed lines show the average proportional evidence where the optimal observer chose to respond whilst the solid lines show the fit of an exponentially decreasing function.* ***b)*** *The same simulated data as in* ***a)*** *is shown with the fit of a power function. The simulations show a steeper decrease than can be captured by the power function (the simulated optimal observer is more cautious with fewer samples and more liberal with more samples).* ***c)*** *The y-axis shows the proportional ideal accumulated evidence ($|\ell_n|$, see equation 4 in manuscript, which describes the decision evidence undisrupted by noise and leak). The dashed lines show the proportional evidence by the number of samples for each confidence rating (three observers in separate plots). The solid lines show the fit of the exponential function.* ***d)*** *Solid lines show the model fitted bounds on the observer's estimated proportional evidence. Dotted lines show the average proportional evidence for each confidence rating. The proportional evidence is the proportional ideal evidence disrupted by noise and leak according to equation 8 of the manuscript. This was estimated using the best-fitting parameters for describing the confidence ratings of the observer.*

The exponential function has three parameters: the scale, b, the rate of decline, λ, and the minimum, a, as described in Equation 9 in the Methods. Although performance is moderated by the combination of these parameters, the observer would need to primarily adjust b to account for inference noise (increasing inference noise requires an increased b to maintain the same accuracy), whilst stronger temporal biases require the adjustment of λ, as illustrated in Supplementary Figure 4**.** The optimal observer primarily adapts the scale, b, and the rate of decline, λ, according to the target performance level. Modelling suggested that observers were adjusting only the rate of decline of their bound, not the scale, in order to adjust their performance according to the target performance in the Stopping task. This selective adjustment of the rate of decline of decision bounds suggests a link between the adaptability of Type-I decision bounds and Type-II sensitivity; perhaps the Type-II system is more capable of accounting for temporal biases than internal noise. Further investigation will be necessary to assess whether this effect is adaptive, or the result of the temporal bias being more accessible to estimate than noise. Previous evidence has suggested that observers show systematic biases in accounting for noise in their confidence judgements[2,3,4], or even blind to the noise affecting their perceptual decisions[4].

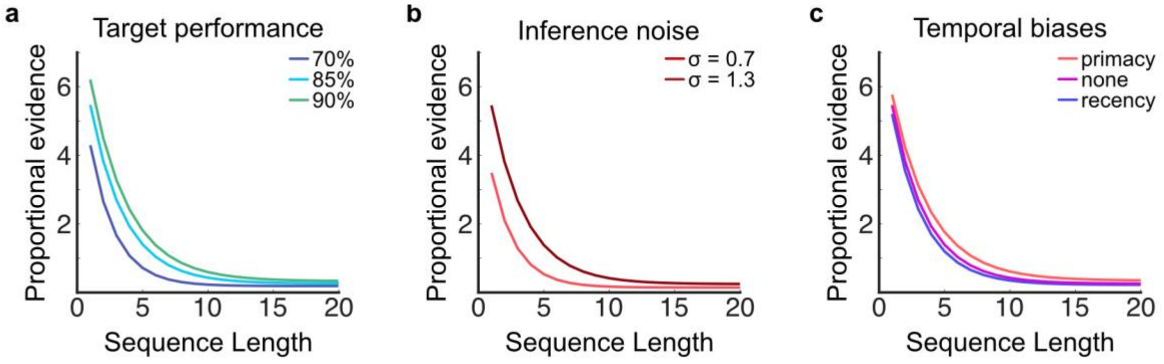

Supplementary Figure 4. Optimal decision bounds. *As performance is moderated both by inference noise and temporal biases, the optimal observer adjusts the parameters of the bound differently for different suboptimalities.* ***a)*** *As the target performance level increases, the optimal observer increases both b and λ.* ***b)*** *An increase in inference noise requires an increase in b (as seen from the height of the lines on the y-axis).* ***c)*** *The optimal observer alters λ to cope with changes in the temporal biases (increasing λ for increasing primacy) as seen from the rate of decline of the lines.*

We suggested that observers were using their confidence evidence to set and maintain the Type-I decision bounds. In this respect, the suboptimalities in confidence evidence are responsible for the suboptimalities in bound efficiency. By simulating optimal bounds on the confidence evidence (for example, stopping the Type-I accumulation when there was a 70% chance of a correct response based on the Type-II evidence), and then re-calculating the bound efficiency based on the accumulated Type-I evidence, we were able to reproduce the relationship between bound efficiency and Type-II

efficiency (y = 1.22x+0.33 p = 0.001; or excluding one outlier, y = 1.6x+1.7, p = 0.008; Supplementary Figure 5a). The simulated bound efficiency was fairly well related to the bound efficiency estimated using observer's actual responses (y = 0.78x+0.08, p = 0.02; Supplementary Figure 5b).

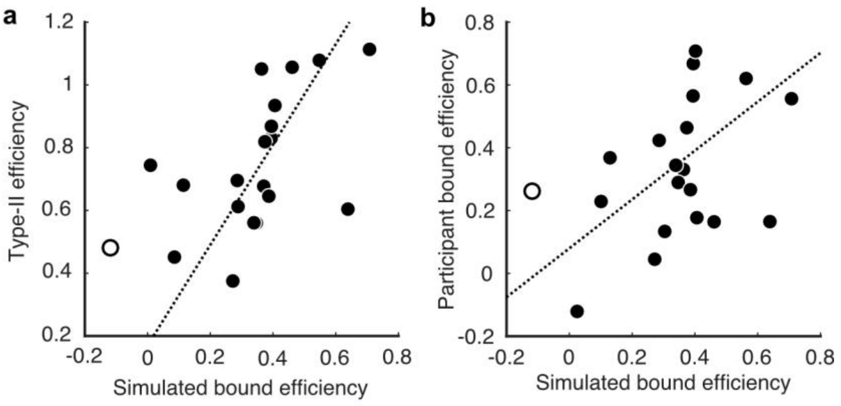

Supplementary Figure 5. Simulated bound efficiency. a) *Bound efficiency was simulated based on parameters fit in the Replay task, by using the Type-II evidence to estimate when to stop accumulating, and then calculating the corresponding bound on Type-I evidence. This provided the same relationship with observers' Type-II efficiency as reported in Figure 3d of the manuscript.* ***b)*** *Relationship between simulated bound efficiency (based on parameters fit to the Replay task) and bound efficiency calculated based on performance in the Stopping task (completed in a different experimental session).*

Supplementary Note 4

Parameter recovery was used to ensure the model fitting procedure was able to accurately recover the underlying parameters describing observers' behaviour. We simulated 500 data sets of 300 trials using parameters sampled from normal distributions centred on the mean parameter values fitted to human observers (Supplementary Table 1). These simulated responses were then fit using the exact same code as for fitting participants' data, and the input (ground-truth) and output (best-fitting) parameters compared. We were particularly concerned about the model's ability to accurately recover the parameters analysed in the Results, given that the likelihood function was numerically estimated based on only 1,000 particles using a noisy objective function[5]. Supplementary Figure 6 shows the simulated and recovered parameters for inference noise (σ), leak (α), and the rate of decline of the bound (λ). We observed limited evidence for systematic biases in the recovered parameters and few large deviations from equality. All parameters showed strong correlations between simulated and recovered values (all $p < 10^{-5}$), indicating that the parameters could be accurately estimated by the model.

<table>
<tr><th colspan="2">Task</th><th>σ</th><th>α</th><th>a</th><th>b</th><th>λ</th><th>μ_U</th><th>σ_U^2</th></tr>
<tr><td rowspan="3">Stopping</td><td>70</td><td rowspan="3">1.01±0.21</td><td rowspan="3">0.99±0.03</td><td rowspan="3">0.11±0.17</td><td rowspan="3">3.83±2.76</td><td>2.17±0.86</td><td rowspan="3">203.75±214.00</td><td rowspan="3">0.64±0.02</td></tr>
<tr><td>85</td><td>3.55±1.20</td></tr>
<tr><td>90</td><td>4.38±1.58</td></tr>
<tr><td colspan="2">Free</td><td>0.87±0.21</td><td>0.93±0.08</td><td>0.1±0.04</td><td>4.73±2.60</td><td>2.74±1.08</td><td>177.8±58.95</td><td>0.64±0.29</td></tr>
<tr><td colspan="2">Replay</td><td>0.67±0.19</td><td>0.77±0.21</td><td>0.16±0.11</td><td>7.22±5.70</td><td>2.64±0.90</td><td>~</td><td>~</td></tr>
</table>

Supplementary Table 1. Average fitted parameters to Type-I behaviour. *For the Stopping task, each condition was first fit separately, the only parameter allowed to significantly differ across conditions was the rate of bound decline, λ. We therefore fit all conditions together, allowing only λ to vary across conditions. The Free task was fit separately, as it was performed in a different session to the Stopping task. In the Replay task, there was no need to fit μ_U and σ_U^2, which describe non-decision time, as observers were not free to stop evidence accumulation and had to wait for a 'go' signal to respond. The noise and leak parameter values fitted in the Replay task appear to differ from values obtained in the Stopping and Free task, suggesting differences in evidence accumulation between conditions where the observer has to also think about when to decide and conditions where the (same) observer has to wait for a 'go' signal. Each entry is the mean and standard error of 20 participants.*

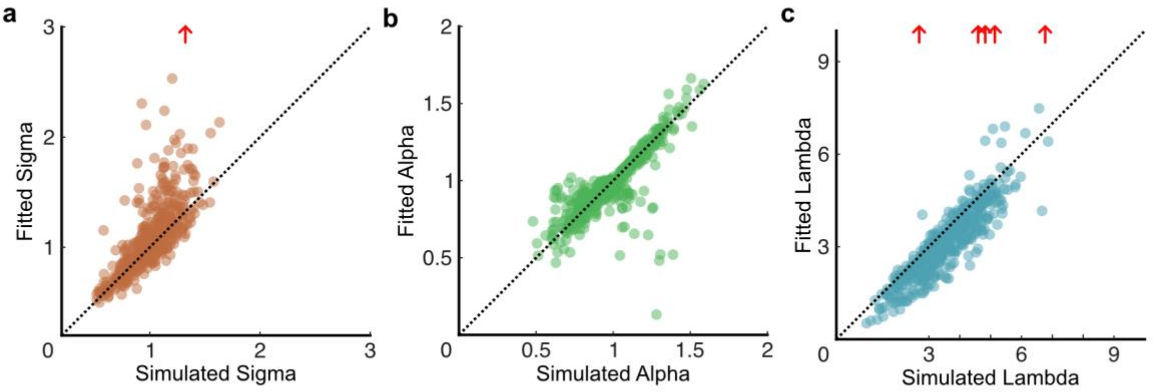

Supplementary Figure 6. Parameter recovery results. *500 simulated data sets were created by simulating responses using random combinations of parameter values that were normally distributed around the mean value fitted to observers' responses. The computational model was then fit to the data to test whether the model would accurately recover the input parameters. We observed a strong correlation between each of the input and output parameters, with limited variability. Here the recovered (best-fitting) parameters are plotted on the y-axis, and the input (ground-truth) parameters on the x-axis,* ***a)*** *for inference noise (σ, $r = 0.83$, $p < 0.001$, uncorrected, two sided),* ***b)*** *leak (α, $r = 0.84$, $p < 0.001$, uncorrected, two sided), and* ***c)*** *the rate of the decline of the bound (λ, $r = 0.90$, $p < 0.001$, uncorrected two-sided). Out of the 500 simulations, one out-of-range data point is not visible in* ***a)*** *and five in* ***c),*** *these are indicated by the red arrows. Importantly, none of the fitted values to the human data were this extreme.*

Supplementary Note 5

When the observer cannot control the amount of evidence they accumulate, they may either: 1. Accumulate all the evidence they are provided and then make their response (no-bound) 2. Establish a covert bound, accumulate evidence to this bound, and then ignore any additional evidence (absorbing bound) or 3. Establish a covert bound, accumulate evidence to this bound, and then continue to monitor any additional evidence in case there is evidence against their decision (reflexive bound[6]). In the results we presented the data comparing the absorbing bound to the no-bound model, but we first compared the three different possible bounds. Supplementary Figure 7 shows the relative fit of the different bounds. There was no evidence that the reflexive bound was a better description of behaviour compared to no-bound ($p = 0.13$), and this bound was a significantly worse fit compared to the absorbing bound ($p_{bonf*3} < 0.006$).

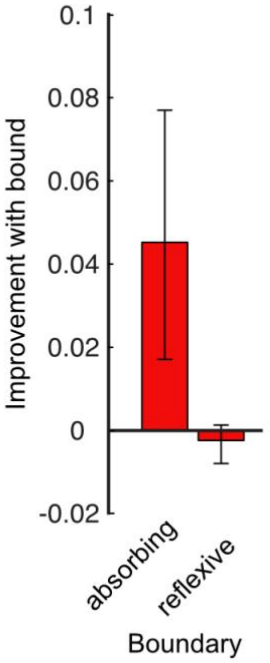

Supplementary Figure 7. Model comparison of different bounds. *The y-axis shows the relative improvement in fit, with the first bar comparing the absorbing bound to the no-bound model, the second comparing the reflexive bound to the no-bound model. Error bars show 95% between-subjects confidence intervals (n=20).*

Supplementary Note 6

In the Results section of the main text, we present the baselined pupil size, z-scored within each observer. Supplementary Figure 8a, and 8d shows the raw z-scored average pupil size and Supplementary Figure 8b and 8e shows the residual data once the predicted pupil size has been removed, based on the impulse response function of the pupil to each stimulus onset, trial start, and eye blinks, using an ARX model[7]. The baselined data are shown in Supplementary Figure 8c and 8f for comparison. In our pre-registration (https://osf.io/gy2t3/) we had included the ARX model as a main analysis because we were concerned that visual stimulation may mask pupil changes driven by cognitive effects (e.g., the crossing of a covert decision bound). The effect of crossing the bound in the Stopping task is clearly visible in the residual pupil responses in Supplementary Figure 8b, and removing the impulse response to the stimulus seems to have aligned the conditions more clearly. However, as shown in Supplementary Figure 8e, the majority of the bound-crossing effects obtained in the Replay task are not visible in the residual. It is possible that the ARX model captured some of the effect as an increase due to visual stimulation, since in the Replay task the timing of the bound crossing overlapped with the timing of the visual stimulation the model was predicting. Given this confound, the ARX model seemed inappropriate for testing our hypotheses. Furthermore, we found the effect of stimulus presentation saturated relatively early in the trial and could be largely counteracted in the response aligned data by simply by baselining the data, as can be seen from comparing Supplementary Figures 8a and 8c. We therefore chose to rely on data baselining in the analyses described in the manuscript.

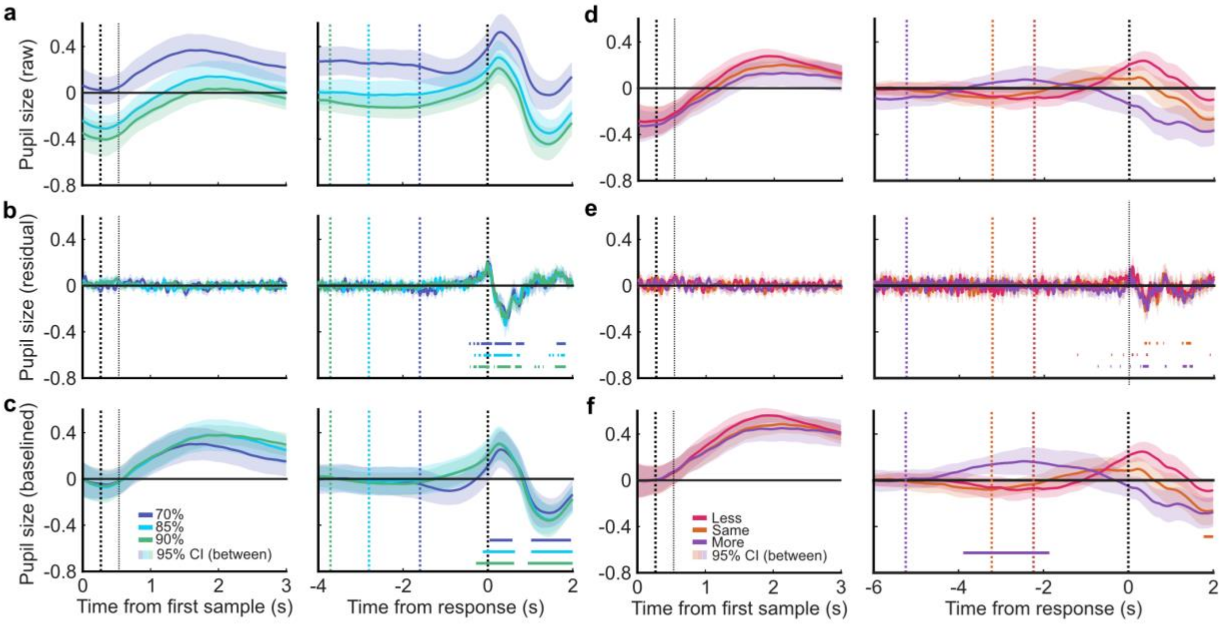

***Supplementary Figure 8. Analysis of pupil size. a)** Pupil size (z-scored) averaged across participants in the Stopping task, without subtracting the baseline from the trial-start aligned data (left) and the response- aligned data (right). The same is shown for the Replay task in **d)**. **b)** Residual pupil size after removing the predicted pupil size based on the impulse response function of the pupil to the trial start, stimulus onset, and blinks, in the Stopping task. The impulse response function was found using an ARX model, following the procedure of Zenon (2017)[7]. The same is shown for the Replay task in **e)**. Plots **c)** and **f)** are shown in Figure 4 of the main results, and show the baseline-corrected data. In all cases, the shaded error bars show the 95% between-subjects confidence intervals.*

A difference in pupil size could be due to a shift in the timing of the pupil response, or driven a difference in the rate of pupil change. In the Results section, we present a difference in pupil size following the response for high confidence compared to low confidence trials. This effect was driven by a more rapid pupil constriction in high confidence trials, based on the difference in the derivatives of the pupil size (i.e. change in pupil size across successive time samples, Supplementary Figure 9a, p <0.002). There was also a significant difference in pupil size between crossed and not crossed trials following the response. Here, it is clear that although the pupil was smaller in crossed trials, the pupil was actually showing faster constriction in not-crossed trials (Supplementary Figure 9b, $p = 0.002$). Finally, we demonstrate that these effects are still visible when dividing trials by both confidence and bound crossing. As shown in Supplementary Figure 9c, there was still an effect of confidence within not-crossed trials ($p_{bonf*4} = 0.024$) and an effect of boundary crossing in high confidence trials ($p_{bonf*4} < 0.004$ and $p_{bonf*4} = 0.024$ for the differences before and after the response respectively). This again supports the finding that two distinct effects in pupil dilation are visible: 1. dilation relative to committing to a type-I decision (which is temporally offset according to different conditions in the Replay task) and 2. more rapid constriction with greater Type-II confidence, following the response.

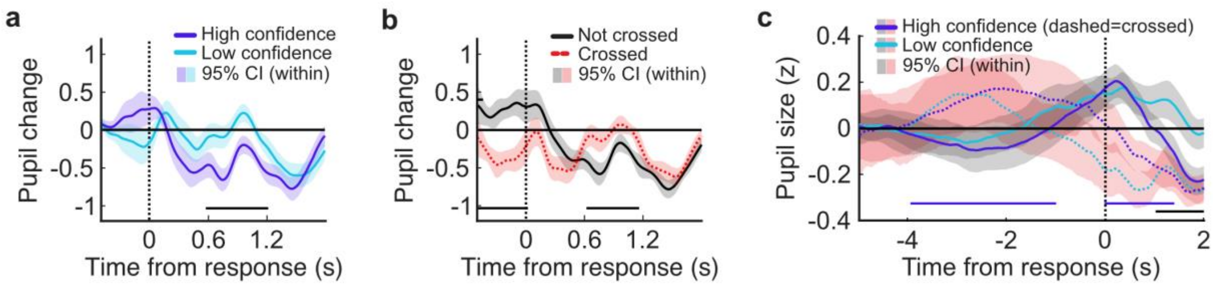

***Supplementary Figure 9. Change in pupil size and analysis within conditions**. a) Change in pupil size in high (dark blue) and low (cyan) confidence trials. The black horizontal line marks the significant cluster, the dashed vertical line shows the time of the response. b) Change in pupil size in crossed (red dashed) and not crossed (black) trials. The black horizontal lines show the significant clusters and the dashed vertical line shows the time of the response. c) Average pupil size within trials grouped by both confidence and bound crossing. Dashed lines with red error bars show crossed trials and black error bars with solid lines show not crossed trials. Dark blue lines show high confidence and cyan lines show low confidence. The black horizontal line shows the significant cluster within not crossed trials (a significant difference between high and low confidence trials). The dark blue horizontal lines show significant differences within high confidence trials (a significant difference between crossed and not crossed trials). In all cases error bars show 95% within-subjects CI.*

References

1 Fleming, S. M., & Lau, H. C. How to measure metacognition. *Frontiers in Human Neuroscience*, **8**, 1–9 (2014). DOI: 10.3389/fnhum.2014.00443

2 Zylberberg, A., Roelfsema, P. R., & Sigman, M. Variance misperception explains illusions of confidence in simple perceptual decisions. *Consciousness and Cognition*, **27**, 246-253 (2014). DOI: 10.1016/j.concog.2014.05.012

3 De Gardelle, V., & Mamassian, P. Weighting mean and variability during confidence judgments. *PloS One*, **103**, e0120870 (2015). DOI: 10.1371/journal.pone.0120870

4 Castañón, S. H., Moran, R., Ding, J., Egner, T., Bang, D., & Summerfield, C. Human noise blindness drives suboptimal cognitive inference. *Nature Communications*, **101**, 1719 (2019). DOI: 10.1038/s41467-019-09330-7

5 Acerbi, L., & Ma, W. J. Practical Bayesian optimization for model fitting with Bayesian adaptive direct search. In *Advances in neural information processing systems* 1836-1846 (NIPS 2017).

6 Zhang, J., Bogacz, R., & Holmes, P. A comparison of bounded diffusion models for choice in time controlled tasks. *Journal of Mathematical Psychology*, **534**, 231-241 (2009). DOI: 10.1016/j.jmp.2009.03.001

7 Zénon, A. Time-domain analysis for extracting fast-paced pupil responses. *Scientific Reports*, **7**, 41484 (2017). DOI: 10.1038/srep41484
