# Supplementary material for: Confidence controls perceptual evidence accumulation
Source: Nat Commun. 2020 Apr 9;11:1753. doi: 10.1038/s41467-020-15561-w (PMC7145794; doi:10.1038/s41467-020-15561-w)
Supplement: Supplementary file 3 — Reporting Summary [file 41467_2020_15561_MOESM3_ESM.pdf]

nature research

Corresponding author(s): Dr Tarryn Balsdon

Last updated by author(s): Oct 24, 2019

Reporting Summary

Nature Research wishes to improve the reproducibility of the work that we publish. This form provides structure for consistency and transparency in reporting. For further information on Nature Research policies, see Authors & Referees and the Editorial Policy Checklist .

Statistics

For all statistical analyses, confirm that the following items are present in the figure legend, table legend, main text, or Methods section.

n/a	Confirmed	
☐	☒	The exact sample size (*n*) for each experimental group/condition, given as a discrete number and unit of measurement
☐	☒	A statement on whether measurements were taken from distinct samples or whether the same sample was measured repeatedly
☐	☒	The statistical test(s) used AND whether they are one- or two-sided<br>*Only common tests should be described solely by name; describe more complex techniques in the Methods section.*
☒	☐	A description of all covariates tested
☐	☒	A description of any assumptions or corrections, such as tests of normality and adjustment for multiple comparisons
☐	☒	A full description of the statistical parameters including central tendency (e.g. means) or other basic estimates (e.g. regression coefficient) AND variation (e.g. standard deviation) or associated estimates of uncertainty (e.g. confidence intervals)
☐	☒	For null hypothesis testing, the test statistic (e.g. *F*, *t*, *r*) with confidence intervals, effect sizes, degrees of freedom and *P* value noted<br>*Give P values as exact values whenever suitable.*
☐	☒	For Bayesian analysis, information on the choice of priors and Markov chain Monte Carlo settings
☒	☐	For hierarchical and complex designs, identification of the appropriate level for tests and full reporting of outcomes
☐	☒	Estimates of effect sizes (e.g. Cohen's *d*, Pearson's *r*), indicating how they were calculated

Our web collection on statistics for biologists contains articles on many of the points above.

Software and code

Policy information about availability of computer code

Data collection	Stimulus presentation and behavioural data collection was performed using Matlab 2016a and the Psychophysics Toolbox (version 3). Pupil data was collected using the EyeLink software.
Data analysis	Data analysis was performed using custom code in Matlab. Code is available from a fork of the pre-registration: https://osf.io/s6zfb/

For manuscripts utilizing custom algorithms or software that are central to the research but not yet described in published literature, software must be made available to editors/reviewers. We strongly encourage code deposition in a community repository (e.g. GitHub). See the Nature Research guidelines for submitting code & software for further information.

Data

Policy information about availability of data

All manuscripts must include a data availability statement. This statement should provide the following information, where applicable:

- Accession codes, unique identifiers, or web links for publicly available datasets
- A list of figures that have associated raw data
- A description of any restrictions on data availability

Data is available from a fork of the pre-registration: https://osf.io/c9xfr/

Field-specific reporting

Please select the one below that is the best fit for your research. If you are not sure, read the appropriate sections before making your selection.

☐ Life sciences ☒ Behavioural & social sciences ☐ Ecological, evolutionary & environmental sciences

Behavioural & social sciences study design

All studies must disclose on these points even when the disclosure is negative.

Study description	This study was designed to examine the relationship between the accumulation of evidence for Type-I and Type-II decisions using behavioural responses, computational modelling and pupillometry
Research sample	Participants were recruited from the RISC mailing list, which is open to anyone wishing to participate in experimental studies. Participants were required to have normal or corrected to normal vision. Twenty participants were included in the analysis, with mean age 24.68 (SD 3.8), 13 were female. The sample is not representative of the world population, the majority were French students. The sample was chosen because it was accessible for the ethical recruitment of human participants.
Sampling strategy	Participants were recruited until 20 full data sets could be included in the analysis. Pre-registered inclusion criteria for analysis was performance above chance. 22 participants were tested with 2 participants showing chance performance and therefore excluded from further analysis. A sample size of 20 was chosen based on previous experiments (the majority of experiments using confidence ratings include 11-40 participants, Rahnev et al., 2020), to enable the detection of a moderate effect size of 0.68 with a power of 0.8 at an alpha level of 0.05 in within-subjects statistical comparisons.
Data collection	Stimuli were presented on a 24-inch LCD monitor (BenQ) running at 60 Hz with a resolution of 1920x1080 pixels and mean luminance 45cd/m2. Stimulus generation and presentation was controlled by MATLAB (Mathworks) and the Psychophysics toolbox4, run on a Mini Mac (Apple Inc). An EyeLink 1000 infrared monocular eye-tracker system (SR Research Ltd. Ontaro, Canada), running at 500 Hz on a dedicated PC, was used to monitor blinks and pupil dilation in the observer's dominant eye. Observers viewed the monitor from a distance of 60 cm, with their head supported by a chin rest. Responses were entered via a standard QUERTY keyboard. All participants completed the same experimental conditions and the experimenter was aware of the experimental design. The first author recruited and tested participants, and was present for instruction and practice, and remained outside the testing room during the experimental session.
Timing	Data were collected between June and July 2018
Data exclusions	Two participants' data were excluded from the analysis based on the pre-registered criteria that their performance did not rise above chance. One participants' pupil data were not included in the analysis due to a technical error where data recording haltted part way through the experiment.
Non-participation	Four participants signed up to the experiment but did not come to the lab for testing due to scheduling errors on their behalf or family emergencies. One participant was unable to participate because their glasses were not compatible with the eye-tracker.
Randomization	Participants were not allocated into different groups. All participants completed the same experiment.

Reporting for specific materials, systems and methods

We require information from authors about some types of materials, experimental systems and methods used in many studies. Here, indicate whether each material, system or method listed is relevant to your study. If you are not sure if a list item applies to your research, read the appropriate section before selecting a response.

Materials & experimental systems

n/a	Involved in the study	
x		Antibodies
x		Eukaryotic cell lines
x		Palaeontology
x		Animals and other organisms
	x	Human research participants
x		Clinical data

Methods

n/a	Involved in the study	
x		ChIP-seq
x		Flow cytometry
x		MRI-based neuroimaging

Human research participants

Policy information about studies involving human research participants

Population characteristics	Participants were 20 human subjects, mean age 24.68 (SD 3.8), 13 were female.
Recruitment	Participants were recruited from the RISC mailing list, which is open to anyone wishing to participate in experimental studies. The majority of subscribers are French tertiary students. This self-selection bias means the sample is unlikely to be representative. We do not expect age, race, gender, level of education, or other social factors to influence how evidence is accumulated for making perceptual and confidence decisions about low-level visual stimuli in normally sighted individuals.
Ethics oversight	Ethical approval was granted by the local ethics committee Conseil d'évaluation éthique pour les recherches en santé (CERES) after a detailed explanation of the experiment was assessed.

Note that full information on the approval of the study protocol must also be provided in the manuscript.
